# Emerging biomarkers for the diagnosis of severe neonatal infections applicable to low resource settings

**Published:** 2011-12

**Authors:** Thor A. Wagner, Courtney A. Gravett, Sara Healy, Viju Soma, Janna C. Patterson, Michael G. Gravett, Craig E. Rubens

**Affiliations:** 1 Seattle Children’s, Seattle, Washington, USA; 2 Department of Pediatrics at University of Washington School of Medicine, Seattle, Washington, USA; 3 Global Alliance to Prevent Prematurity and Stillbirth, an initiative of Seattle Children’s, Seattle, Washington, USA; 4 Seattle Biomedical Research Institute, Seattle, Washington, USA; 5 Department of Obstetrics and Gynecology, University of Washington, Seattle, Washington, USA

## Abstract

More than 500 000 children die each year in low resource settings due to serious neonatal infections. Better diagnostics that can be utilized in these settings to identify infected infants have the potential to significantly reduce neonatal deaths and the associated morbidity. A systematic review was performed and identified more than 250 potential new biomarkers for the diagnosis of serious neonatal infections. Eight of these biomarkers were both high-performance and high-abundance (antithrombin, inter-α inhibitor proteins, interferon-γ inducible protein-10, interleukin-1 receptor antagonist, LPS binding protein, mannose binding lectin, serum amyloid A, resistin, visfatin), and are promising for the diagnosis of serious neonatal infections in low resource settings. Future clinical trials comparing these biomarkers with more traditional biomarkers seem warranted.

Reducing global childhood mortality by two-thirds is a Millennium Development Goal of the United Nations. Severe neonatal infections are one of the most significant causes of pediatric mortality, resulting in more than 500 000 deaths each year (1). 99% of these deaths occur in low resource settings (2). Identifying neonates with severe infections is difficult in high resource settings, and limited laboratory capability in low resource settings makes diagnosis even more challenging. Clinical criteria for the diagnosis of neonatal ‘sepsis’ have been developed and are included in the WHO Integrated Management of Childhood Illness (IMCI) program (3). In one large multicenter study of neonates seeking medical attention in low resource settings, the ICMI guidelines were 85% sensitive and 75% specific (4). There are increasing efforts to have community health care workers visit all newborns and implement interventions according to IMCI guidelines (5). As more neonates are screened for severe neonatal infections, the predictive value of the clinical guidelines would be expected to decrease, resulting in a much larger percentage of misdiagnoses, with significant associated mortality, cost, and complications. Inexpensive point-of-care testing that could increase the performance (both sensitivity and specificity) of these diagnostic algorithms has the potential to substantially improve the global management of severe neonatal infections.

This review sought to identify promising new biomarkers for the diagnosis of serious neonatal infections, characterize the biomarkers with the greatest potential utility in low resource settings, and help prioritize biomarkers that warrant further research and/or development. We focused on the performance of soluble biomarkers and combined biomarkers. Hundreds of biomarkers were identified that have been associated with ‘sepsis’ or predicted to be good biomarkers for sepsis. This review focused exclusively on biomarkers with published performance data for the diagnosis of serious neonatal infections. New biomarkers whose performance appears to have the potential to outperform existing biomarkers are highlighted. Because there are theoretical benefits to combined biomarkers, and because combined biomarkers are becoming increasingly feasible in less expensive point-of-care formats, additional effort was made to identify the performance of biomarker combinations.

## METHODS

### Literature review strategy

**Figure 1:**
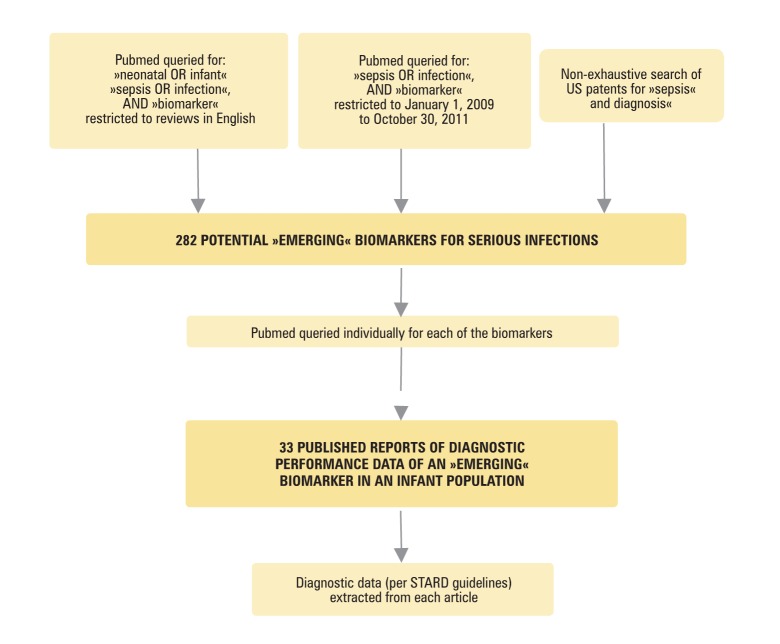
Strategy used to identify individual »emerging« biomarkers for the diagnosis of serious neonatal infections. Procalcitonin, C-reactive protein, tumor necrosis factor-α, interferon-γ, interleukin-6, and interleukin-8, were considered »established« biomarkers and were not reviewed.

This search was focused on identifying “emerging” soluble host response biomarkers for the diagnosis of serious newborn infections. Biomarkers for the diagnosis of serious newborn infections that have been studied extensively in high resource settings such as procalcitonin (PCT), C-reactive protein (CRP), tumor necrosis factor-α (TNF-α), interferon-γ (IFN-γ), interleukin-6 (IL-6), and interleukin(IL-8), have been reviewed elsewhere (6-8) and were not the focus of this review, unless they were included in a combined biomarker panel. Several different strategies were utilized to identify a broad list of potential biomarkers for the diagnosis of serious neonatal infections. First, Pubmed was queried for “neonatal OR infant”, “sepsis OR infection”, and “biomarker” (**Figure 1**) The search was restricted to reviews in English, which identified 119 abstracts. Ninety-four abstracts were focused exclusively on existing biomarkers. Thirteen review articles were relevant to novel or emerging biomarkers, and reviewed in detail to identify potential biomarkers for severe neonatal infections (6,7,9-19). Second, more relaxed searches not restricted to neonates or infants, or not restricted to reviews, but published within the last two years, were also performed in an effort to identify emerging biomarkers, e. g., references (20-31). Third, a non-exhaustive search of US patent and patent applications using www.uspto.gov and www.google.com/patents using ‘sepsis’ and ‘diagnosis’ identified other potential biomarkers, e. g. references (32,33). This combined search strategy identified 282 potential biomarkers. Starting with this broad list of potential biomarkers, Pubmed was searched to identify original research articles regarding each of these biomarkers. Thirty-three studies provided diagnostic performance data in infant populations (20,24,30,34-63). Pubmed was also queried for “neonatal OR infant”, “sepsis OR infection”, and “combination biomarker” to identify 19 studies that evaluated the performance of combinations of biomarkers for the detection of serious newborn infections (25,29,35,51,52,57,64-79). When necessary, corresponding authors were contacted to clarify aspects of the respective studies.

### Data collected

Positive predictive value (PPV) and negative predictive value (NPV) were felt to be clinically relevant metrics but were hard to compare across studies in which disease prevalence differed. Sensitivity and specificity are independent of disease prevalence and easier to compare across studies. Area under curve (AUC) of the receiver operator characteristic (ROC) curve is a widely used summary measure of diagnostic assay performance (80). Data on these performance parameters was collected when present or when it could be calculated from the published data. Standards for Reporting of Diagnostic Accuracy (STARD) represent expert opinion regarding 25 items that should be included in diagnostic literature (81). Data on these performance characteristics was collected if available.

### Biomarker performance characteristics of interest for low resource settings

In order to help identify biomarkers that would improve the performance over existing clinical algorithms, performance data was considered promising if sensitivity or specificity was greater than 90%, and/or AUC>0.9. Technical features of the assay applicable to implementation in low resource settings were also evaluated. Specifically, we sought biomarkers that appeared promising for adaptation to low cost point-of-care formats. Turn-around time of less than two hours and the ability to perform the test without laboratory infrastructure have been considered essential features for implementation in the lowest resource settings (82). Currently, lateral flow immunoassays are the primary diagnostic format that meets these criteria. Lateral flow immunoassays in widespread clinical use generally have a lower limit of detection (LOD) of 1ng/mL, although newer methods, such as a Europium-based lateral flow assay with a LOD of 0.3ng/mL, have been reported (83). None of the new biomarkers described in this review were tested in a lateral flow format, but we focused on relatively high abundance biomarkers (≥1ng/mL), that could theoretically be adapted to a lateral flow format with existing technology. Because the precision of inexpensive lateral flow tests is usually decreased, good discrimination between the limit of detection and the diagnostic cut-off was also considered important for assay performance. Testing cord blood was felt to be impractical on a large scale in low resource settings, and performance data on biomarkers that were only tested on cord blood were not included. Other characteristics of the biomarker that seemed to have potential to impact their use in low resource settings were also noted.

## RESULTS

### Summary of biomarkers identified

In recent years, genomic and proteomic technology has identified numerous gene transcripts and proteins associated with ‘sepsis’, and increasing understanding of immune responses has led to many proposed biomarkers for sepsis. The majority of these biomarkers have not been evaluated as diagnostics, and only a few of those have been studied in children. We were able to identify infant diagnostic performance data on 23 biomarkers. Seven of these biomarkers were acute phase reactants (serum amyloid A (SAA) (19,51,58-62), LPS binding protein (LBP) (41-45), inter-α inhibitor proteins (IαIp) (46,47), antithrombin (48-50), and soluble E-selectin (20,51,52), fibronectin(pFN) (52-54), and mannose binding lectin (MBL) (30) (**Table 1**).

**Table 1:** Acute phase reactant biomarkers for neonatal sepsis

Biomarker Name	Sample Size(n)	Sensitivity (%)	Specificity (%)	PPV (%)	NPV (%)	Cut-off Level	LOD	ROC (AUC)	Assay method	Reference
**Serum Amyloid A (SAA)**	192	24	93	67	67	1 mg/mL	Not given	0.61	Automated immunoassay	51
	163	76.4	100	100	58	6.8mg/100mL	6.8mg/100mL	0.88	Immune-nephlometric assay	58
	134	96 (0 h) 96 (24 h)	95 (0 h) 98 (24 h)	85 (0 h) 92 (24 h)	99 (0 h) 99 (24 h)	8mg/L (0 h) 10mg/L (24 h)	0 to 386 μg/mL	0.99 (0 h) 0.99 (24 h)	Latex photometric immunoassay	61
	116	95 (0 h) 100 (8 h) 97 (24 h)	93 (0 h) 85 (8 h) 77 (24 h)	87 (0 h) 76 (8 h) 67 (24 h)	97 (0 h) 100 (8 h) 98 (24 h)	10 μg/mL	Not given	0.81 (0 h) 0.81 (8 h)	ELISA	59
	94	100	93	96	Not given	10 μg/mL	Not given	Not given	ELISA	60
	40	Not given	Not given	Not given	Not given	N/A	4ng/mL	0.94	ELISA	57
	46	98	92	90	98	41.3mg/L	Not given	0.98	nephlometry	62
**LPS Binding Protein (LBP)**	140	80	55	Not given	Not given	26.6 μg/mL	0.2 μg/mL	0.82	Chemiluminescent immunoassay	41
	46	82 (0 h) 85 (24 h)	86 (0 h) 86 (24 h)	65 (0 h) 66 (24 h)	94 (0 h) 95 (24 h)	11.4mg/L (0 h) 17.2 mg/L (24 hr)	0.2mg/L	0.86 (0 h) 0.91 (24 h)	Chemiluminescent immunoassay	42
	96	100 (age <48 hr) 92 (age >48 hr)	94 (age <48 hr) 89 (age >48 hr)	89 (age <48hr) 66 (age >48hr)	100 (age <48 hr) 92 (age >48 hr)	21.5 mg/L (age <48 hr) 17.1 mg/L (age >48 hr)	Not given	0.97 (age <48 hr) 0.93 (age >48 hr)	Chemiluminescent immunoassay	43
	60	97	70	37	92	12.7 mg/L	Not given	0.90	Chemiluminescent immunoassay	44
	69	not evaluated, but levels of LBP in septic neonates were significantly elevated compared to healthy neonates (median, 36.6 vs 7.8 μg/mL)					0.45 μg/mL	Not evaluated	Enzyme immunoassay	45
**Inter-alpha Inhibitor Proteins (IαIP)**	573	90	99	95	98	177 mg/L	50 mg/L	0.94	ELISA	46
	135	Not evaluated	Not evaluated	Not evaluated	Not evaluated	Not evaluated	100 mg/L	Not evaluated	ELISA	47
**Antithrombin (AT)**	60	Not evaluated, but antithrombin functional levels were in septic neonates were significantly decreased compared to controls (mean, 34.87% vs. 90.5%, *P*<0.001)					Not given	Not evaluated	ELISA	48
	150	Not evaluated, but antithrombin functional levels in septic neonates were significantly lower than controls					Not given	Not evaluated	Colorimetric assay	49
	58	92	62	60	93	15 mg/dL	Not given	Not given	Not given	50
**Soluble E-selectin**	192	55	82	67	74	132 ng/mL	0.009 ng/mL	0.72	ELISA	51
	120	59	87	81	69	130 ng/mL	0.5 ng/mL	0.8	ELISA	52
	108	Not evaluated				Not given	Not given	Not evaluated	Antibody microarray	20
**Fibronectin (pFN)**	65	100	87.5	Not given	Not given	90 μg/mL for preterm 100 μg/mL for term	Not given	Not given	ELISA	53
	58	74	74	58	85	Not given	Not given	Not given	Not given	54
	220	75	63	11	98	120 μg/mL (age <34 wk) 145 μg/mL (age >34 wk)	Not given	Not given	ELISA	55
**Mannose Binding Lectin (MBL)**	97	96.7	97.1	98.3	94.4	0.5 μg/mL	0.075 μg/mL	Not given	immunoassay	30

PPV – positive predictive value, NPV – negative predictive value, ROC (AUC) – receiver operator curve (area under the curve), LOD – lower limit of detection, ELISA – enzyme-linked immunosorbent assay

Fourteen cytokine biomarkers were identified, including six pro-inflammatory cytokines (interleukin-1α(IL-1α) (34), interleukin-1β(IL-1β)(35,36), interleukin-12p70(IL-12p70) (35,36), interleukin-18(IL-18) (20,37), granulocyte colony stimulating factor(G-CSF) (38,39), and resistin (24); two anti-inflammatory cytokines – interleukin-10(IL-10) (73) and interleukin-1 receptor antagonist(IL-1RA) (39,40); one probable cytokine – visfatin (24); and five chemokines – growth related oncogene α(GRO-a) (35), interferon-γ-inducible protein 10(IP-10) (35,84), monokine induced by interferon-γ(MIG) (35), regulated upon activation normal T cells expressed and secreted(RANTES) (35), and monocyte chemoattractant 1(MCP-1) (35) (**Table 2**).

**Table 2:** Emerging cytokine and chemokine biomarkers for neonatal sepsis

		Biomarker name	Sample size	Sensitivity (%)	Specificity (%)	PPV (%)	NPV (%)	Cut-off	LOD	ROC (AUC)	Assay method	Reference
**CYTOKINES**	**Proinflammatory**	Interleukin-1α (IL-1α)	62	83	88	77	92	12 pg/mL	1 pg/mL	Not given	ELISA	34
		Interleukin-1β (IL-1β)	155	50	63	25	76	3.7 pg/mL	7.2 pg/mL	0.57	Cytometric bead assay	35
			164	11	97	40	87	90 pg/mL	Not given	0.61	Cytometric bead assay	36
		Interleukin-12p70 (IL-12p70)	155	64	56	36	80	2.7 pg/mL	1.9 pg/mL	0.62	Cytometric bead assay	35
			164	28	98	71	89	75 pg/mL	Not given	0.74	Cytometric bead assay	36
		Interleukin-18 (IL-18)	84	Not available, IL-18 levels increased in septic neonates vs. neonates with necrotizing enterocolitis				Not given	12.5 pg/mL	Not given	ELISA	37
			108	Not evaluated, but IL-18 levels were highly associated with sepsis				Not given	Not given	Not given	ELISA	20
		Granulocyte colony stimulating factor (G-CSF)	171	95	73	40	99	200pg/mL	10 pg/mL		ELISA	38
			254	57	95	86	82	950 pg/mL	Not given	0.8	ELISA	39
		Resistin	105	93	95	Not given	Not given	8 ng/mL	1.85 ng/mL	0.91	EIA	24
		Visfatin	105	92	94	Not given	Not given	10 ng/mL	Not given	0.92	EIA	24
	**Anti-inflammatory**	Interleukin-10 (IL-10)	155	84	84	67	93	7.6 pg/mL	3.3 pg/mL	0.90	Cytometric bead assay	35
		Inteleukin 1 receptor antagonist (IL-1ra)	101	93	92	Not given	Not given	11 000 pg/mL	440 pg/mL	0.94	2-step ELISA	40
			254	33	89	80	82	Not given	Not given	0.73	ELISA	39
**CHEMOKINES**	**CXC**	Growth related oncogene-α (GRO-α)	155	82	60	45	89	55 pg/mL	10 pg/mL	0.81	Cytometric bead assay	35
		Interferon-γ-inducible protein-10 (IP-10)	155	93	89	77	97	1250 pg/mL	2.8 pg/mL	0.95	Cytometric bead assay	35
			60	81	95	Not given	Not given	48 pg/mL	1.67 pg/mL	0.84	ELISA	63
		Monokine induced by IFN-γ (MIG)	155	80	78	59	91	650 pg/mL	2.5 pg/mL	0.84	Cytometric bead assay	35
	**CC**	Regulated upon Activation Normal T cells expressed and Secreted (RANTES)	155	86	45	38	89	11 800 pg/mL	10 pg/mL	0.67	Cytometric bead assay	35
		Monocyte chemoattractant protein 1 (MCP-1)	155	68	68	46	84	357 pg/mL	2.7 pg/mL	0.78	Cytometric bead assay	35

PPV – positive predictive value, NPV – negative predictive value, ROC (AUC) – receiver operator curve (area under the curve), LOD – lower limit of detection, ELISA – enzyme-linked immunosorbent assay, EIA – enzyme immunoassay

One soluble cell surface marker, soluble intercellular adhesion molecule-1(sICAM-1) (51,56) and one molecule involved in triglyceride metabolism, apolipoprotein CII (ApoC2) (57) were also identified (**Table 3**). Although no absolute performance data are available for interleukin-18 (IL-18), it was included based on the strength of performance data relative to other biomarkers (20,37).

**Table 3:** Diagnostic performance of other biomarkers for neonatal sepsis

Biomarker name	Sample size (n)	Sensitivity (%)	Specificity (%)	PPV (%)	NPV (%)	Cut-off	LOD	ROC (AUC)	Assay method	Reference
**Soluble intercellular adhesion molecule 1 (sICAM-1)**	149	77	76	67	84	228 ng/mL	0.096 ng/mL	0.79	ELISA	51
	43	88	86	75	94	300 ng/mL	Not given	Not given	Not given	56
**Apolipoprotein C2 (ApoC2)**	80	Not evaluated				Not given	0.3 ng/mL	0.79	Mass spectrometry and ELISA	57

PPV – positive predictive value, NPV – negative predictive value, ROC (AUC) – receiver operator curve (area under the curve), LOD – lower limit of detection, ELISA – enzyme-linked immunosorbent assay

### Summary of individual biomarker performance

For the 23 soluble biomarkers with published diagnostic performance data in infant populations, the available data regarding sensitivity, specificity, PPV, NPV, and area under receiver operator curves is summarized in Tables 1, 2 and 3. The collective performance of these biomarkers varied widely: sensitivity from 11–100%, specificity from 45–98%, PPV from 35–96%, NPV 66−98%, and area under the receiver operator curve of 0.57–0.95. There was often significant variability in the performance of individual biomarkers when evaluated in separate studies. To assess the technological feasibility of these assays in low resource settings, assay method, limit of detection, and cut-off concentration were also recorded. All of the soluble biomarkers were measured by immunoassay, most by enzyme linked immunossorbent assays, although some newer studies were done with cytometric bead assays and/or using chemiluminesence. One study used an unbiased proteomics approach to identify promising biomarkers that were then quantified by immunoassay (57). None of these assays were performed in a point-of-care format. The cut-off concentrations used for the cytokine biomarkers (2.7 pg/mL − 12 ng/mL) were orders of magnitude lower than the acute phase reactants (130 ng/mL – 177 mg/mL).

### Most promising individual biomarkers

Despite the limitations of the data, nine soluble biomarkers (IL-1ra, IP-10, SAA, LBP, IαIp, resistin, visfatin, MBL, and AT) emerged as promising individual candidates for further study (**Table 4**).

**Table 4:** Diagnostic performance of the most promising biomarkers for neonatal sepsis

Biomarker name	Class	Assay method	No. of neonatal studies	Median study size (range)	Median sensitivity (range)	Median specificity (range)	Median PPV (range)	Median NPV (range)	Median AUC (range)	Median cut-off (range)	References
**IL-1ra**	Cytokine	2-step ELISA or ELISA	2	176 (101-254)	63 (33-93)	91 (89-92)	80	82	0.84 (0.73-0.94)	11.5 ng/mL (11-12 ng/mL)	39,40
**IP-10**	Chemokine	ELISA or cytometric bead assay	2	108 (60-155)	87 (81-93)	92 (89-95)	77	97	0.95	0.649 ng/mL (0.048-1.25 ng/mL)	35,63
**Resistin**	Adipocytokine	EIA	1	105	93	95	Not given	Not given	0.91	8 ng/mL	24
**Visfatin**	Adipocytokine	EIA	1	105	92	94	Not given	Not given	0.92	10 ng/mL	24
**SAA**	Acute phase reactant	Latex agglutination or ELISA	7	104 (40-192)	96 (24-100)	93 (92-100)	89 (67-100)	97 (58-99)	0.91 (0.61-0.99)	25.7 µg/mL (8-1000 µg/mL)	51,57-62
**LBP**	Acute phase reactant	Chemiluminescence or enzyme or immunoassay	4	65 (46-140)	92 (80-100)	86 (55-94)	66 (37-80)	96 (92-100)	0.90 (0.82-0.97)	17.1 µg/mL (11.4-26.6 µg/mL)	41-44
**IαIP**	Acute phase reactant	ELISA	1	573	90	99	95	98	0.94	177 µg/mL	46
**MBL**	Innate pattern recognition	Immunoassay	1	97	97	97	98	94	Not given	0.5 µg/mL	30
**AT**	Anticoagulation	Not given	1	92	92	62	60	93	Not given	150µg/mL	50

IL-1ra – interleukin (IL)-1 receptor antagonist, IP-10 – interferon g-induced protein 10, SAA – serum amyloid A, LBP – lipopolysaccharide-binding protein, IαIp – inter-alpha inhibitor protein, MBL – mannose-binding lectin, AT – antithrombin, – positive predictive value, NPV – negative predictive value, ROC (AUC) – receiver operator curve (area under the curve), ELISA – enzyme-linked immunosorbent assay

IL-1ra and IP-10 are both inflammatory cytokines that are elevated early in infection (85,86). The best reported sensitivity of IL-1ra (100%) is promising but the range of reported sensitivities (33%-100%) is concerning. IL-1ra has a short half-life of 4 to 6 hours (87) which may explain the variability in sensitivity and could be a limitation for general use as a stand-alone biomarker for severe neonatal infections. Studies of IP-10 have shown moderate sensitivity (81%-93%) despite significant difference in cut-off concentrations (1.2 – 48 ng/mL). One study demonstrated an excellent AUC (0.95), which may be the single most important performance parameter. The immune physiology of IP-10 is also attractive because although it is a chemokine it is interferon-induced like other acute phase reactants, with the potential benefit of assessing different aspects of the immune response.

The physiologic roles of resistin and visfatin are less well characterized. Resistin was initially described as an adipocyte-secreted peptide (adipokine) but is now known to be secreted by monocytes and to be a more general pro-inflammatory cytokine (88). Visfatin was also initially described as an adipokine and an insulin mimetic. However, visfatin is also known as pre-B cell colony-enhancing factor (PBEF), which is a cytokine that is increased in a variety of inflammatory conditions and can induce cellular expression of other pro-inflammatory cytokines, such as TNF-a, IL-1b, and IL-6 (89). In one report, both molecules performed well as biomarkers for serious newborn infections, with sensitivity and specificity greater than 90%. The cut-offs of 8 ng/mL and 10 ng/mL respectively, should be easily achievable in a lateral flow format (24). Despite the relatively limited amount of performance data these molecules appear promising and seem to warrant further study.

The five remaining promising biomarkers are all acute phase reactants (SAA, LBP, IαIp, MBL, AT). Acute phase reactants are attractive biomarkers for severe neonatal infections because they are usually produced in large quantities by the liver for a relatively long duration. This makes them easier to quantify and provides a wider time window during which they are useful as biomarkers. Because their production is regulated by the cytokine response, the acute phase reactants tend to be produced slightly later in the course of infection (90). Therefore, compared to cytokines, acute phase reactants may be less effective diagnostic biomarkers at earlier stages of infection.

Serum amyloid A (SAA) is probably the single most promising biomarker. SAA performed extremely well in four studies published by three different groups (57,60-62) (sensitivity 96%-100%, and ROC AUC of 0.94 – 0.997), and performed reasonably well in a fifth study, with a sensitivity of 76% and a ROC AUC of 0.875 (58). In contrast to these five studies, one study showed relatively poor performance with a sensitivity of 24%, and ROC AUC 0.61, although the specificity was 93% (51). The cut-offs used in these studies varied considerably, from 0.8 mg/L to 1000 mg/L. However, the three studies that used a cut-off of 50mg/L or less showed good sensitivity. The study by Ng et al (57) also showed good performance, and although they did not report a specific cut-off for SAA, based on the range of values in the septic children vs controls a cut-off between 11-15mg/L would have had no overlap between SD of the two populations. This data suggests that SAA is a robust biomarker for the diagnosis of serious newborn infections, although the cut-off concentration is critical for its diagnostic performance.

Despite its name, LBP is elevated in both gram-negative and gram-positive infections, and has at least moderate sensitivity (80−100%), as reported by multiple groups (41,43-45). IαIP also performed relatively well (sensitivity 90%) in the largest study (n=573), which was well-designed and prospective (46). The NPV was 97%, which may be an important performance characteristic if the biomarker is used as a screening test for severe neonatal infections. Mannose-binding lectin (MBL) is also a promising biomarker. MBL plays an important pattern recognition role in the innate immune response to pathogens, triggering the eponymous MBL pathway to complement cascade activation (91). In one recent study (30) MBL had a sensitivity of 97% and specificity of 97% for the diagnosis of septic preterm and term neonates. Antithrombin (AT) is another molecule that seems to have potential as a biomarker for serious neonatal infections. AT has anticoagulant activity and is consumed during serious infections (49). AT has been associated with sepsis in three studies (48-50), and performed reasonably well in the one study which reported diagnostic performance, with a sensitivity of 92% and a NPV of 93%, although the specificity was only 62% (50). IαIp, MBL, and AT are noteworthy because they decrease during infection, which makes them potentially very attractive to use in combination with other biomarkers that increase during infection.

Two other biomarkers seem intriguing and may have potential utility in the diagnosis of serious neonatal infections, and therefore seem worth noting. G-CSF is a key cytokine in the canonical neutrophil response to severe bacterial infections that should rise before more classical markers of infection (e. g. white blood cell and band counts), making it a logical potential biomarker for early detection of infection (92). While G-CSF did perform reasonably well in two studies, it is present at relatively low concentrations (<1 ng/mL), making adaptation to a point-of-care format challenging. ApoC2 was originally associated with preterm sepsis in a study by Rovamo et al in 1984 (93) and was more recently identified by Ng et al (56) in an unbiased proteomic screen as a potential biomarker of severe neonatal infections. ApoC2 is synthesized by the liver and is involved in triglyceride synthesis, but its role in infection remains speculative. In the validation phase of the study by Ng et al (57), ApoC2 did not perform well alone (ROC curve area 0.79), but was identified through logistic regression as an optimal biomarker when combined with SAA (ROC curve area 0.96).

### Combination biomarkers

Currently available analyses of combination biomarkers have been rudimentary and have had mixed results (35,51,52,57,64-79). One exception was the recent study by Ng et al (57) which had a more sophisticated proteomic-based biomarker discovery phase, followed by logistic regression to identify optimal biomarker combinations, and performance was validated in a separate cohort. **Table 5** summarizes data about the performance of combination biomarkers. The majority of these studies have evaluated biomarkers in combination with CRP because CRP is already in widespread clinical use for the diagnosis of infection. CRP is less useful in the earliest phases of severe neonatal infection because it is an acute phase reactant and does not peak until 12 to 24 hours after infection and can also be triggered by non-infectious insult such as trauma (68). Recent studies have shown that the diagnostic performance of CRP may be improved upon when used in combination with other acute phase reactants and early mediators of inflammation. A study by Dollner et al (66) compared the diagnostic performance of CRP, IL-6, soluble tumor necrosis factor p55 and p75, soluble ICAM-1 and soluble (s) E-selectin. CRP was the best single test with a sensitivity of 70% and specificity of 90%, but sensitivity or specificity could be improved when combined with IL-6. Another study that evaluated levels of sICAM-1, sE-selectin and SAA in combination with CRP found that combining all four biomarkers increased sensitivity from 70% for CRP alone to 90%, but specificity remained low at 67% (51). Hansen et al observed that the sensitivity and NPV of CRP were significantly improved when combined with sICAM-1 levels. In neonates under 5 days old, sensitivity increased from 69% to 93% and NPV increased from 73% to 92% (68). Not all studies have demonstrated improved diagnostic utility when biomarkers are combined. Resch et al evaluated the reliability of procalcitonin (PCT), IL-6 and CRP to diagnose early onset neonatal sepsis and found that combining the best performing marker, PCT, with either IL-6 or CRP did not increase the sensitivity for diagnosing sepsis compared to using PCT alone (78).

**Table 5:** Diagnostic performance of combination biomarkers

Biomarker(s)	Sample size (n)	Notes	Sensitivity (%)	Specificity (%)	PPV (%)	NPV (%)	Cut-off value	Reference
CRP	192	*reported for culture positive cases	80	71	43	93	0.4mg/L	51
CRP+sICAM-1, SAA, and sE-selectin			85	67	94	41	0.4mg/L, 249ng/mL, 1mg/L, 132mg/L	
CRP+ sICAM-1			72	75	44	91	0.4mg/L, 249ng/mL	
CRP		*reported for culture positive and culture negative, clinically suspected sepsis cases	69	70	60	79	0.4mg/L	
CRP+sICAM-1, SAA, and sE-selectin			90	67	64	91	0.4mg/L, 228ng/mL, 1mg/L, 132mg/L	
CRP+ sICAM-1			79	76	68	85	0.4mg/L, 228ng.mL	
								
sTREM-1	52		70	71	62	78	144pg/mL	75
IL-6			80	81	74	86	66pg/mL	
sTREM-1 + IL-6			90	62	81	77	144pg/mL, 66pg/mL	
								
PCT	98		65	60	52	59	63.4 pg/mL	77
IL-10			92	84	80	89	17.3 pg/mL	
nCD64			92	71	69	83	2.6%	
IL-10 + nCD64			95	83	79	86	17.3 pg/mL, 2.6%	
PCT + IL-10			75	68	53	64	36.4 pg/mL, 17.3 pg/mL	
PCT + nCD-64			78	64	58	69	36.4 pg/mL, 2.6%	
								
ApoC2/SAA	104	*at day 0	96	76	82	95	0.199	57
		*day 0 and day 1	100	61	75	100	0.199	
								
CRP	120		86	97	96	88	8 mg/L	52
sE-selectin			59	87	81	69	130 ng/mL	
CRP + sE-selectin			45	100	100	65	8 mg/L, 130 ng/mL	
								
PCT	123		21	92	46	79	25 ng/mL	64
			69	67	39	87	5.75 ng/mL	
IL-6			57	94	76	88	250 pg/mL	
			71	71	43	89	12 pg/mL	
PCT + IL-6			71	88	65	91	25ng/mL, 250 pg/mL	
								
IP-10	155		93	89	77	97	1250 pg/mL	35
IP-10 +IL-6			98	72	58	99	1250 pg/mL, 26.1 pg/mL	
IP-10 + IL-6 + IL-10			98	61	50	99	1250 pg/mL, 26.1 pg/mL, 7.6 pg/mL	
IP-10 + IL-10			98	76	61	99	1250 pg/mL, 7.6 pg/mL	
IP-10 + GRO-α			96	58	47	97	1250 pg/mL, 55 pg/mL	
IP-10 + GRO-α +IL-8			100	39	39	100	1250 pg/mL, 55 pg/mL, 62 pg/mL	
IP-10 + IL-8			98	53	45	98	1250 pg.mL, 62 pg/mL	
IP-10 + IL-8 + MIG			98	44	41	98	1250 pg.mL, 62 pg/mL, 650 pg/mL	
IP-10 + MIG			93	76	60	97	1250 pg/mL, 650 pg/mL	
IP-10 + GRO-α + IL-10			98	51	44	98	1250 pg/mL, 55pg/mL, 7.6pg/mL	
IP-10 + GRO-α + MIG			96	47	42	96	1250 pg/mL, 55 pg/mL, 650 pg/mL	
								
CRP	77		96	89	79	98	5 mg/L	70
IL-6			35	93	67	76	150 pg/mL	
CRP + IL-6			35	100	100	78	5 mg/L, 150 pg/mL	
CRP and/or IL-6			96	82	69	98	5 mg/L, 150 pg/mL	
								
CRP	111		65	52	63	54	14 mg/L	76
CRP + IL-6			92	41	67	80	14 mg/L, 60 pg/mL	
CRP + IL-8			97	41	68	92	14 mg/L, 50 pg/mL	
								
								
CRP	1291		54			86	10 mg/L	68
IL-8			44	90	58	83	70 pg/mL	
CRP + IL-8			80	87	68	93	10 mg/L, 70pg/mL	
								
CRP	359	*at 0 hours	49	91	73	77	10 mg/L	67
CD64			79	89	78	89	6136 antibody-PE molecules bound/cell	
CRP + CD64			81	82	69	89	10 mg/L, 6136 antibody-PE molecules bound/cell	
CRP		*at 24 hours	60	83	64	80	10 mg/L	
CD64			96	81	71	97	6136 antibody-PE molecules bound/cell	
CRP + CD64			97	71	73	98	10 mg/L, 6136 antibody-PE molecules bound/cell	
								
CRP	105	*at 0 hours	69	96	93	80	10 mg/dL	71
IL-6			76	73	67	81	18 pg/mL	
IL-8			75	66	60	80	100 pg/mL	
CRP + IL-6			89	73	70	90	10 mg/dL, 18 pg/mL	
CRP + IL-8			89	66	65	90	10 mg/dL, 100 pg/mL	
CRP		*at 24 hours	78	94	91	83	10 mg/dL	
IL-6			63	76	74	66	18 pg/mL	
IL-8			49	79	71	59	100 pg/mL	
CRP + IL-6			83	78	75	84	10 mg/dL, 18 pg/mL	
CRP + IL-8			76	79	79	83	10 mg/dL, 100 pg/mL	
								
CRP	76		49	100	100	58	8 mg/L	78
PCT			77	91	93	72	6 ng/mL	
PCT + CRP			83				8 mg/L	
PCT + IL-6			89				8 mg/L	
								
CRP	60		80	92			1.52 mg/dL	74
CRP + IL-8 +sReceptor IL-2			85	97			1.52 mg/dL, 63 pg/mL, 2780U/mL	
								
CRP	110	*at 0 hours	65	99	96	87	12 mg/L	73
IL-6			78	92	81	91	31 pg/mL	
CD64			95	88	80	97	4000 PE molecules bound/cell	
CRP		*at 24 hours	72	100	100	90	12 mg/L	
IL-6			44	93	72	81	31 pg/mL	
CD64			97	90	80	99	4000 PE molecules bound/cell	
CRP(0 hr) + IL-6(0 hr) + CD64(24hr)			100	86	74	100	12 mg/L, 31 pg/mL, 4000 PE molecules bound/cell	
								
CRP	166		63	89			10 mg/L	66
IL-6			78	64			20 pg/mL	
CRP +/or IL-6			96	58			10 mg/mL, 20 pg/mL	
								
CRP	90		69	86	84	73	5 mg/L	69
sICAM-1			78	90	90	80	300µg/mL	
CRP + sICAM-1			93	80	82	92	5 mg/L, 300µg/mL	
								
CRP	101		60	100	100	75	12 mg/L	79
IL-6			89	96	95	91	31 pg/mL	
TNF-α			82	86	82	85	17 pg/mL	
CRP + IL-6			93	95	95	95	12 mg/L, 31 pg/mL	
CRP + TNF-α			91	86	84	92	12 mg/L, 17 pg/mL	
IL-6 + TNF-α			95	84	83	96	31 pg/mL, 17 pg/mL	
CRP + IL-6 + TNF-α			95	84	82	96	12 mg/L, 31 pg/mL, 17 pg/mL	
								
IL-6	55		80	78			500 pg/mL	65
TNF-α			73	94			70 pg/mL	
IL-6 + TNF-α			60	100			500 pg/mL, 70 pg/mL	
								

CRP – C-reactive protein, IL – interleukin, sICAM -1 – soluble intercellular adhesion molecule-1, SAA – serum amyloid A, IL-12p70 – IL-12 protein 70, sTREM-1 – soluble triggering receptor expressed on myeloid cells, ApoC2 – apolipoprotein C-II, GRO-a – growth-related oncogene-a, MIG – monokine induced by interferon g, PCT – procalcitonin, TNF – tumor necrosis factor, PPV – positive predictive value, NPV – negative predictive value, PE – phycoerythrin

Luminex, mass spectrometry, and other highly multiplexed detection methods have allowed for increased screening of biomarker combinations in the last several years. In a 2007 study, Ng et al (35) associated elevated levels of interferon-γ-inducible protein 10 (IP-10) with neonatal sepsis. As mentioned earlier, using IP-10 levels alone resulted in a sensitivity and specificity of 93% and 89%, respectively, with a NPV of 97%. When IP-10 concentration was combined with various other markers of infection such as IL-6, IL-8, and IL-10, the sensitivity and NPV were slightly improved by up to 7%, but the specificity and PPV were dramatically decreased by up to 50% (35). In 2010 Ng et al (57) reported an unbiased, mass spectrometry-based, proteomic approach to identify biomarkers that were specifically associated with acute neonatal sepsis and normalized after treatment. Not only did they identify a previously undescribed biomarker (Pro-ApoC2), but they also used logistic regression to identify a combination of two biomarkers (Pro-ApoC2 and SAA) that resulted in a test with 96% sensitivity and 76% specificity. The combined ApoSAA score had a NPV of 95% on day 0 (of suspected infection) and 100% when levels were measured on days 0 and 1. Early detection of infection based on the combined biomarkers could potentially result in a 45% reduction of antibiotic use when antibiotic therapy is withheld or discontinued in uninfected infants (57). The experimental approach used to identify this combination required advanced technology and rigorous mathematical analysis, but both biomarkers are present at relatively high levels and should be amenable to a multiplexed lateral flow format making the ApoSAA score an extremely promising combined biomarker.

A few studies report on the combined use of soluble biomarkers with flow cytometry to measure cell surface receptor expression. An early study by Ng et al (73) in 110 neonates found that combining IL-6 and CRP levels measured at 0 hours with CD64 measured at 24 hours yielded good diagnostic performance with sensitivity, specificity, PPV and NPV of 100, 86, 74, 100%. CD64 measured at 24 hours performed almost as well on its own with 97% sensitivity and 90% specificity (73). A follow-up study in 2004 by Ng et al (67) again showed improved diagnostic performance of CRP and CD64 together vs CRP alone (sensitivity increased from 49% to 81%), but the excellent performance of the earlier study was not replicated, and the performance of the combined biomarker did not outperform CD64 alone. Zeitoun et al (77) evaluated the performance of CD64 in combination with IL-10 and found that the combined biomarker had a sensitivity of 95% and specificity of 79%, but the combination did not perform significantly better than IL-10 alone. Although CD64 is promising alone or in combination, quantification requires measuring the mean fluorescent intensity of individual cells, which diminishes the feasibility of this approach in low resource settings.

## DISCUSSION

This review identified at least nine biomarkers (AT, CRP, IαIp, IL-1ra, IP-10, SAA, LBP, MBL, PCT, resistin, visfatin) that appear promising for the diagnosis of serious neonatal infections in low resource settings. These biomarkers appear to have better performance than the existing clinical algorithms used in low resource settings. Furthermore, the clinical cut-off concentration used for these biomarkers were all in a range that should be detectable with lateral flow immunassays, a diagnostic technology platform that has a proven track-record in low resource settings. Especially with further study of these biomarkers in combination, there seems to be great potential to improve the diagnosis of severe neonatal infections in low resource settings.

Although these emerging biomarkers are promising, there are important limitations to the current literature. All of the studies reviewed focused on severe neonatal infections, yet there was significant heterogeneity in how this population was defined. Some studies excluded premature or low birth-weight infants, the populations most vulnerable to infection. “Neonatal” included infants ranging from birth to two months old. The definition of ‘sepsis’ was also quite variable, particularly in instances of suspected sepsis with negative blood cultures and whether coagulase negative staphylococcal growth in a blood culture was considered sepsis. Timing of diagnostic testing relative to the onset of symptoms was also variable. Importantly, none of these assays were tested in low-resource settings, where rates of inflammation and/or the pre-test probability of infection may be different from high resource settings. The heterogeneity of the studies makes it difficult to compare the relative performance of biomarkers across studies. Furthermore, many of the studies did not compare the performance of new biomarkers to established biomarkers (e. g. CRP), which makes their benefit relative to existing biomarkers difficult to assess. The performance data for many of the biomarkers comes from a single study, for example with IαIp, MBL, resistin, visfatin. Where multiple published reports of a marker exist, they often come from a single research group. Given the large number of biomarkers reported to be associated with ‘sepsis’, reporting bias is a concern. For such biomarkers, confirmation of performance in additional studies, preferably by other research groups will be particularly important in order to help validate the performance of these biomarkers. In contrast, a few biomarkers, like SAA, LBP, and IP-10, have shown consistently good performance in several studies, and are more likely to be reliable diagnostic biomarkers.

Another potential limitation of the data are that most of the studies reviewed included relatively small numbers of participants (average population size of 135) and over-fitting of the biomarker performance is a significant concern. In almost all of the studies reviewed the diagnostic cut-off was fit to the data set, often using receiver operator curves, and therefore likely represents the best-case performance for the biomarker. All of the reviewed biomarkers should be considered to be at the discovery phase and will need independent cross-validation to accurately evaluate their performance. One potential exception is the 2010 study by Ng et al that used a more rigorous approach, starting with unbiased proteomics to discover mass spectrometry peaks associated with sepsis, then refining that set of potential biomarkers by focusing on peaks that showed a reversal pattern after resolution of sepsis. These peaks were then identified and quantified, and logistic regression was used to identify the combination of biomarkers with the most discriminatory power. A score based on this combined biomarker was cross-validated in an independent case-control group as well as a prospective cohort (57). This robust approach is much more likely to identify biomarkers and cut-offs that are reproducible in future studies.

Despite the limitations noted above, several soluble biomarkers seem to have potential to significantly improve the diagnosis of severe neonatal infections. The number of studies reflects not only the perceived clinical need for better diagnostics but also a significant amount of work that has already been done on biomarker discovery. In contrast, less effort has been directed toward determining the optimal combinations of biomarkers and validating previously identified biomarkers. Theoretically, a combination of these biomarkers should have the best performance. However, the number of potential biomarker combinations rises exponentially, where the number of possible combinations = 2*^p^*^ − 1^, and *p* is the number of biomarkers. Because the number of participants in a study should theoretically be greater than the number of biomarker combinations evaluated, much larger studies will be necessary to identify and validate combination biomarkers. This imposes practical limits on the total number of biomarkers that can be analyzed, but it seems feasible to validate 5−10 of the biomarkers identified in this review, in combination with a few of the promising traditional biomarkers. To be relevant, future studies should be conducted in low resource settings, with careful definition of ‘sepsis’, consideration for the amount of blood that can routinely be obtained, and designed with significant biostatistical guidance.

Severe neonatal infections are a significant cause of global mortality. Modest improvement in the diagnosis of severe neonatal infections could lead to significant decreases in infant mortality and a substantial number of lives saved. The actual impact of diagnostics depends on the availability and performance of the test, as well as the availability, uptake, and effectiveness of the treatment based on the test results. Large numbers of small studies have described hundreds of biomarkers associated with severe neonatal infections. The aim of this review was to summarize and consolidate the extensive work that has been already been done with the hope of helping to prioritize biomarkers that warrant further study. Large rigorous validation studies focusing on combinations of the most promising biomarkers (CRP, PCT, IL-1ra, IP-10, SAA, LBP, MBL, IαIp, AT, resistin, visfatin, and perhaps G-CSF and ApoC2) are necessary in order to determine their true performance characteristics and seem warranted in an effort to reduce global infant mortality.
